# Supplementary material for: How Australian Rural Health Academic Centres Contribute to Developing the Health Workforce to Improve Indigenous Health: A Focused Narrative Review
Source: Healthcare (Basel). 2025 Aug 1;13(15):1888. doi: 10.3390/healthcare13151888 (PMC12346149; doi:10.3390/healthcare13151888)
Supplement: Supplementary file 1 [file healthcare-13-01888-s001.zip › healthcare-3708850-supplementary.pdf]

Supplementary File S1: Summary of UDRH publications (2010-2021) about Indigenous issues related to the health workforce

Author (Year) Location	Category	Method and Study design	Study population	Study aim	Key findings	Recommendations
Bennett-Levy, J., et al. (2014) Multiple states	Indigenous health workforce	Qualitative Participatory action research	5 Indigenous counsellors	Investigates Indigenous counsellors' views on the appropriateness and efficacy of Cognitive Behavioural Therapy (CBT) for individuals in Australia's Indigenous communities.	Indigenous counsellors trained in CBT found it effective in addressing psychological distress among Indigenous Australian clients.	Further research should focus on integrating CBT into Indigenous mental health frameworks. Practitioners should approach CBT use with sensitivity, guided by client preferences and their own assessments of its appropriateness.
Bennett-Levy, J., et al. (2015) Multiple states	Indigenous health workforce	Qualitative Participatory action research	5 Indigenous counsellors	Investigates how Indigenous counsellors utilised Cognitive Behavioural Therapy (CBT) techniques on themselves during training.	CBT was found beneficial for counsellors, enhancing their skills and lowering stress levels, shielding them from job-related stress and burnout.	Further research to determine what elements of CBT are most effective in the regard to decreasing burnout. CBT training may be beneficial in the Indigenous councillors and health workers daily lives and strengthen their learning of CBT.
Bennett-Levy, J., et al. (2017) NSW	Indigenous health workforce	Mixed methods Interviews and reports on consultation sessions	21 Indigenous and 5 non-Indigenous health professionals.	Examines the uptake of e-Mental Health (e-MH) within Indigenous communities by training healthcare professionals.	Non-credentialed Indigenous health providers in support or educational roles might find greater benefit in training on using e-MH resources for health promotion rather than coaching-based web therapy programs or apps.	To better serve Indigenous communities, culturally, role, and organizationally relevant e-MH resources are necessary. For Indigenous health providers, resources need to expand beyond e-therapy to encompass health education and informational tools for health promotion.
Bennett-Levy, J., et al. (2021) NSW	Indigenous health workforce	Qualitative Meeting notes, written feedback and interviews	Indigenous community leaders and Indigenous health workers	Documents the transformation of a project from a top-down government funded digital mental health training project to a community guided digital social and emotional wellbeing (d-SEWB) training project.	No one place for Indigenous-specific d-SEWB resources. Key learnings were: 1. Community Involvement Is Central to Effective Outcomes; 2. Providing Adequate Funding and Timelines Enables Meaningful Community Input; 3. Set out to Create Co-Learning Hubs; 4. Project Outcomes Are Greatly Enhanced by Building Local Capacity; 5. Time for Reflection Needs to Be Intrinsic to CBPR	Engaging community can change the nature of the work delivered but it enabled successful advocacy and expansion of the range and type of digital resources plus a dedicated “one-stop-shop” d-SEWB website

Author (Year) Location	Category	Method and Study design	Study population	Study aim	Key findings	Recommendations
					Processes; 6. Advocacy Is an Important Component of CBPR Processes.	
Biles, J., et al. (2021) NSW	Indigenous health workforce	Qualitative Interviews	Participants enrolled in a cultural mentorship program aimed at assisting Indigenous nurses and midwives	Investigates how Indigenous nurses from diverse backgrounds in a rural healthcare setting experienced a mentoring program.	Establishing culturally inclusive workplaces involves embracing diversity, providing mutual support, and introducing culturally sensitive initiatives. Supporting Indigenous nurses and midwives in healthcare demands ongoing programs that recognise and promote culturally suitable assistance, ultimately increasing recruitment and retention.	Culturally informed mentoring builds trust and cultivates safe workplaces. Further research is necessary to comprehend its impact on long-term workforce stability and to develop mutually effective mentoring approaches.
Bird, J., et al. (2017) NSW	Indigenous health workforce	Qualitative Interviews	16 Indigenous service providers who had participated in an e-Mental Health (eMH) training program	Studies the use of eMH tools by Indigenous service providers post an eMH training program, endeavouring to identify the specific eMH resources employed in their practice.	Participants utilised eMH resources for diverse purposes such as promoting social inclusion, education, assessment, managing cases, referrals, crisis response, and self/family care. They selected a range of eMH resources, including culturally specific and mainstream options, for client use. Though they directed clients to online treatment programs, they exclusively utilised eMH resources designed for mobile devices during in-person sessions.	The eMH field must broaden its scope beyond primary health care professionals in clinical settings to include community-based workforces outside the conventional health system.<br>It is essential to create culturally appropriate resources, services, and treatments that promote emotional wellbeing and address mental health issues.
Cosgrave, C., et al. (2017) NSW	Indigenous health workforce	Qualitative Interviews	5 Aboriginal mental health workers (AMHWs)	Study explored why AMHWs working in rural and remote NSW community mental health (CMH) services commonly experienced low levels of job satisfaction, especially while undertaking an	The health sciences qualification obtained was not translating into NSW Health's 'professionalised' workplace. Three aspects negatively impact the job satisfaction of AMHWs: (1) difficulties being accepted into the team and organisation; (2) culturally	To rectify conditions that conditions that contribute to job dissatisfaction among rural and remote-based AMHWs. NSW Health could 1). make changes to the degree qualification obtained under the training program 2).raise the level of understanding about the CMH program and Indigenous cultural awareness

Author (Year) Location	Category	Method and Study design	Study population	Study aim	Key findings	Recommendations
				embedded training program.	specific work challenges; and (3) professional differences and inequality.	generally among CMH staff and NSW Health.
Crouch, A., et al. (2020) National	Health workforce	Mixed methods Survey (closed-ended and open-ended questions)	131 health practitioners and students from a range of disciplines (allied health, nursing pharmacy, psychology, social work)	Explores health practitioner knowledge and beliefs regarding Indigenous health and compares with reported burden of disease and health systems data.	Disconnect between health practitioner knowledge of Indigenous health issues and published disease burden. Duration of cultural awareness training impacts effectiveness - less than one full day ineffective. Importance of social and professional relationships between health practitioners and Indigenous Australians.	Pre-service exposure to basic population health science and health systems theory should be embedded in undergraduate curricula for all health disciplines. At least one full day of cultural awareness training required to effectively change attitudes and beliefs. Importance of intentionally building social and professional relationships between health practitioners and Indigenous Australians.
de Witt, A., et al. (2018) Qld	Health workforce	Qualitative Interviews	26 health professionals - 11 Indigenous and 15 non-Indigenous health professionals	Investigates the perspectives of health professionals regarding delivering follow-up cancer care for Indigenous patients.	Essential components for supporting Indigenous cancer survivors' post-treatment involve culturally sensitive care, mental health aid for patients and families, evaluating patient needs, providing logistical assistance (e.g., transportation, lodging), coordinating hospital visits, and advocating for Indigenous health. Indigenous healthcare providers are crucial in ensuring these needs are addressed across primary care settings and in the transition between primary care and hospitals.	Indigenous healthcare workers require adequate support and training, along with the implementation of diverse strategies for culturally sensitive cancer care and addressing workforce shortages. To overcome these shortages, it's crucial to introduce effective pathways that encourage Indigenous participation and completion of higher education in health-related fields.
Dunbar, T., et al. (2019) NT	Health workforce	Qualitative Interviews and focus groups	5 Managers, 29 Remote Area Nurses (RANs), 12 Indigenous staff and 56 community residents	Investigates how the RAN workforce and staffing issues were perceived by clinic managers, RANs, Indigenous colleagues	Participants thought having the "right" nurse was more important than having more nurses. Participants highlighted the need for RANs to have advanced clinical and cultural	Cultural skills and clinical skills must be prioritised in recruitment of RANs. Retention strategies need to be more targeted.

Author (Year) Location	Category	Method and Study design	Study population	Study aim	Key findings	Recommendations
				and community members.	skills.<br>Managers and, to a lesser extent, RANs prioritised clinical skills, while Indigenous staff and community residents prioritised cultural skills.	
Francis-Cracknell, A., et al. (2019)<br>Not applicable	Student workforce	Systematic review	Systematic review of 17 articles	Reviews the impact of Indigenous health curriculum on entry-level health professionals in delivering equitable health care.	Most studies described face-to-face delivery along with blended learning combining a placement in an Indigenous setting, stand-alone placements and digital learning. Five domains of learning: remembering, understanding, self-knowledge, perspective and application relating mostly to cultural awareness.<br>Positive learner reactions were affected by attitude, environment, educator skill, pedagogy and opportunities.	Understanding more about how well graduates are prepared to work in Indigenous health and how to measure this is needed. There is also opportunity to know more about Indigenous health teaching and learning across learning domains, in mainstream clinical placements and in digital learning. Further understanding of educator preparedness is also required.
Harvey, P., et al. (2021)<br>Vic	Student workforce	Quantitative Survey	106 Medical students in the final three clinical years of their degree	Investigate the perceptions of medical students attending a Rural Clinical School on their preparedness to work in the Indigenous Health Context. Explore the students' perceptions of what would improve preparedness.	Half of the students wanted more training about Indigenous peoples' health in the curriculum. 71% of students reported teaching had made them more prepared for practice but 29% still felt unprepared.<br>Self-reported preparedness to work with Indigenous people influenced by: (1) previous educational experience, (2) clinical experience with Indigenous people, (3) teaching by Indigenous peoples and (4) teaching directly delivered within the medical course.	Creating an academic environment that embraces Indigenous knowledge and practices, and ensuring research with Indigenous peoples is underpinned by Indigenous knowledge, can develop enhanced ways of educating medical students.

Author (Year) Location	Category	Method and Study design	Study population	Study aim	Key findings	Recommendations
Isaacs, A. N., et al. (2016) Vic	Student workforce	Quantitative Survey	220 student nurses	Explored cultural desire (as a prerequisite for developing cultural competence) among student nurses towards Indigenous peoples and its association with participation in a one-semester unit on Indigenous health.	Completing the Indigenous Health and wellbeing unit did not influence students' opinions on inclusion of the unit as part of the nursing curriculum or their overall cultural desire. Students who completed the unit reported a higher understanding of Indigenous health but lower interest levels in the subject.	Further research is necessary to explore how and when cultural desire might develop in nurses who are trained in cultural competence particularly in the contexts of post-colonial disparities and political conflict
Jacob, E., et al. (2016) Vic	Student workforce	Quantitative Survey	246 students in various stages of a three-year nursing degree.	Explores content expectations of nursing students required to undertake Indigenous health studies as part of a Bachelor of Nursing Degree at a rural university campus.	Content expectations included Indigenous culture (pre 30.4%–post 29.8%), Indigenous health issues (pre 20.0%–post 23.7%) and understanding nursing care related issues (pre 15.7%–post 17.1%). Data findings were significant for enhancing the current unit. Students expressed the need for a safe learning environment in which to challenge beliefs and opinions.	Course content needs to be sensitive to student populations and include content relevant to the programme being studied. Future research could look at the value of cultural immersion experiences and their ability to improve student confidence in nursing Indigenous people.
Jones, MP et al. (2021) NT	Health workforce	Quantitative NT Department of Health workforce and patient outcomes data analysis	25,413 Indigenous patients and staff from 48 NT government primary healthcare clinics in remote communities	Examines the effect of staff turnover and use of short-term staff in remote clinics in the NT on quality of care for Indigenous patients. Attempts to identify clinic-specific factors which may counter reduced quality of care.	Quality of care received by Indigenous clients varied considerably across indicators and clinics. Higher quality of care more likely for management of chronic diseases and least likely for general adult health checks. Minimal evidence that increased turnover, decreased stability and increased reliance on temporary staff had a negative impact on quality of care.	Clinic-specific factors may counter any potential negative effects of decreased staff employment, however more research is needed to determine what they may be.

Author (Year) Location	Category	Method and Study design	Study population	Study aim	Key findings	Recommendations
Khalil, H. (2019) Vic	Indigenous health workforce	Quantitative Survey	17 Indigenous Health Practitioners	Details the implementation of a medication safety program, while also evaluating it and providing recommendations for future initiatives.	The data analysis showed a notable, meaningful shift in the nurse's understanding, assurance, conduct, and usage concerning medication safety. The program succeeded by identifying crucial problems, engaging local Indigenous Health Practitioners and Indigenous health experts in material development, and establishing a strong, supportive structure within the healthcare organization.	Work together with Indigenous Health Practitioners, experts, and rural health organizations to create effective technologies and policies and build a skilled and well-equipped healthcare team.
Lai, G., et al. (2018) Not applicable	Indigenous health workforce	Systematic review	Systematic review of 15 articles	Identifies factors affecting the retention of Indigenous Australians in the healthcare sector and outlines recommendations and strategies to support the growth of the Indigenous health workforce.	Enhancing culturally supportive work environments, clearly outlining roles and responsibilities, and providing adequate support and remuneration can enhance the retention of Indigenous health professionals.	Up-to-date, national data on the Indigenous healthcare workforce is needed to understand retention rates.<br>Research is required on factors influencing Indigenous people into health careers, as well as strategies to retain Indigenous clinicians.<br>Intervention studies are needed to identify the most effective approaches.
Lenthall, S., et al. (2011) National	Health workforce	Quantitative Survey and database analysis	349 nurses in very remote locations	Describes the registered nursing workforce in very remote Australia and looks at changes over the last 13 years.	The registered nursing workforce in very remote Australia is mostly female and ageing. 43% work in remote Indigenous communities.<br>Few nurses have postgraduate remote qualifications.<br>Over the last decade, the number of nurses per population had fallen; the numbers of midwives and child health nurses had dropped significantly. These trends will have a negative effect on	No recommendations

Author (Year) Location	Category	Method and Study design	Study population	Study aim	Key findings	Recommendations
					Indigenous health outcomes if they continue.	
Lindeman, M., et al. (2014) NT	Health workforce	Qualitative Interviews	33 remote health professionals	This paper analyses data from two qualitative studies to consider current and improved approaches to preparing and supporting staff for conducting aged care and cognition assessments in remote and Indigenous settings.	Themes related to the assessment workforce, current approaches to preparing assessment staff, and cross-cultural knowledge/skill. Critical reflection and cultural safety require the inclusion of cultural supervisors in practitioners' supervision models, and in follow-up. There was general unpreparedness among practitioners for assessment of Indigenous clients.	There is a need for structured approaches to providing practitioners with feedback on both content and the processes of 'reflection-in-action'. Cultural supervisors should be incorporated in supervision models. Communities of practice (online and face-to-face) may assist in professional development and staff retention. Professional support needs to come from management and/or organisations that understand both the context of practice and the nature of clinical practice required and this should be articulated and supported by policy.
Modderman, C., et al. (2020) Vic	Health workforce	Qualitative Interviews	13 social workers (SW) who were overseas-born and educated	Explores the experience of overseas born and educated SWs and their understandings of Indigenous communities.	Majority of overseas born SWs had limited understanding of Indigenous culture. Recruiting organisations were not proactive in dismantling incorrect assumptions. Cultural competency training during induction was confusing and inadequate.	Overseas born SWs need to increase their awareness of differences in their personal lived experiences from Australian Indigenous peoples. Practice of cultural humility may reduce harm. Critical reflection may help counter-colonial thinking in child protection practice.
Nelson, J. R., et al. (2015) Multiple states	Indigenous health workforce	Qualitative Participatory action research	5 Indigenous counsellors	Identifies challenges in supervising mental healthcare workers from Indigenous communities and suggests new supervision approaches.	Current supervision practices are inadequate and lead to high levels of stress and burnout in the Indigenous mental healthcare workforce. Indigenous Health Practitioners require culturally sensitive, safe, and effective clinical supervision to address stress and burnout. This includes access to support designed to	Worker support is an investment rather than an unnecessary cost. Multiple alternative supervision approaches were suggested, including the dual supervision model, cultural and community education, skill development consultation, communities of practice, and supervisor training.

Author (Year) Location	Category	Method and Study design	Study population	Study aim	Key findings	Recommendations
					the unique challenges faced by Indigenous mental health professionals within their communities.	
Plater, S., et al. (2020) Multiple states	Indigenous health workforce	Qualitative Interviews	26 mature-age Indigenous university graduates.	Examines how various forms of structural racism are interconnected, working to authorise, restrain, and manage educated Indigenous people. These systems also create divisions within communities, establishing semi-hierarchical structures, all while upholding white power and advantage.	How these variations exhibit as lower expectations, discrimination based on skin tone, cultural bias, and safeguarding of advantages. These actions can create an invisible barrier for the career advancement of Indigenous individuals, making it challenging for many graduates to move up professionally.	Non-Indigenous Australians must address the distance required to genuinely include Indigenous people as equals. This division isolates these groups from full participation in Australian society and impedes the nation's progress toward embracing future equality.
Plater, S., et al. (2018) Multiple states	Indigenous health workforce	Qualitative Interviews and participant observation	Mature-age Indigenous university graduates	Describes a journey to explore the experiences mature-age Indigenous university graduates and students, as well as the process of decolonising constructivist grounded theory methodology.	Participants shifted the post-graduation narrative from one of personal and professional uplift to one dubbed 'the blessings and burdens of being an educated black'. The participants are activists in the fight for indigenous self-determination.	None.
Prout, S., et al. (2014) WA	Student workforce	Qualitative Student journal entries	27 students from allied health and nursing schools	Examines the experiences of health science students during 'Country Week' - a one-week intensive experiential interprofessional education program in rural Western Australia.	This educational experience provides students with grounded opportunities to develop into effective and reflective practitioners, which is critical for rural health practice. Even for students who do not relocate to regional areas it is essential that they understand the issues that affect access to health among rural and/or Indigenous populations.	The Country Week experience contributes to preparing a health workforce that is ready for the rural health practice

Author (Year) Location	Category	Method and Study design	Study population	Study aim	Key findings	Recommendations
Rae, K., et al. (2016) NSW	Student workforce	Qualitative Interviews and written feedback	6 student and new-graduate dietitians	The study aimed to evaluate the cultural experiences of student and new-graduate dietitians visiting an Indigenous ArtsHealth centre through a quality assurance project.	The themes 'building rapport' and 'developing cultural understanding' were identified from participants experiences. Four participants felt they gained a deeper understanding of the context around health disparity for Indigenous Australians through their experiences.	Students should complete in-depth cultural training before undertaking a placement of this type. Cultural awareness lectures should be as interactive as possible, incorporating practical advice for building rapport and addressing nutrition issues.
Russell, D. J., et al. (2017) NT	Health workforce	Quantitative NT Department of Health payroll and financial dataset analysis	470 nurses, 93 Aboriginal health practitioners (AHPs), 583 other staff employed at 53 remote NT clinics	Measures turnover and retention of health staff in remote NT communities.	NT government-funded remote clinics are small, experience very high turnover and make considerable use of agency nurses. Clinics employed a median of 2 nurses, 0.6 AHPs, 2.2 other staff and 0.4 additional agency nurses. Turnover rates for nurses and AHPs combined was 128%. Only 20% of nurses and AHPs remained working at a specific remote clinic 12 months after commencing. Half left within 4 months.	The NT and Australian governments need to invest in implementing health workforce policies and workforce models that stabilise the remote primary care workforce and reduce turnover. Suggestions include: 1 month on, 1 month off in shared position; preferential selection of rural and remote students into nursing and AHP training courses; remote scholarships; recruitment and retention incentives for working remotely; eliminate barriers to entering health workforce for Indigenous Australians living in remote communities.
Ryder, C., et al. (2017) Multiple states	Student workforce	Quantitative Questionnaire	40 participants - 22 undergraduate students, 9 postgraduate students and 9 staff from the Faculty of Medicine, Nursing and Allied Health	A research questionnaire to measure transformative unlearning, cultural safety and critical thinking in Indigenous Health was developed and tested on undergraduate and postgraduate students and faculty staff. The tool met good standards of reliability and validity.	The initial validation process revealed some concepts with insufficient internal validity. Adjustments will be made to the tool before trialling with additional tertiary institutes to further develop and strengthen the validity and reliability measures of this questionnaire	Validating and improving the questionnaire can assist educators to evaluate their approaches to cultural safety pedagogy and help improve outcomes from Indigenous health and cultural safety training in Australia.

Author (Year) Location	Category	Method and Study design	Study population	Study aim	Key findings	Recommendations
Shahid, S., et al. (2018)<br>Multiple states	Indigenous health workforce	Qualitative<br>Interviews	46 participants - 20 program staff and 26 clinical placement participants (managers, Indigenous Health Workers)	Evaluates and explores experiences with the Program of Experience in the Palliative Approach (PEPA) clinical placements for Indigenous Health Workers (IHWs), aimed at improving understanding of and care delivery in palliative care.	PEPA placements increased participants' confidence in conversations about end-of-life care and facilitated relationships and ongoing work collaboration with palliative care services.<br>Management support was critical, and placements undertaken in settings which had more experience caring for Indigenous people were preferred.	Training for Indigenous participants requires: (1) resources for meaningful engagement with Indigenous communities (e.g., time dedicated to community engagement); (2) cultural safety of participants (e.g., provision of an Indigenous mentor); (3) culturally appropriate resources and (4) consideration for participants across Australia.
Smith, K., et al. (2017)<br>Qld	Health workforce	Mixed methods<br>Survey (closed-ended and open-ended questions)	78 participants - 11 primary healthcare clinicians, 13 other staff, 54 Indigenous consumers	Compares staff and Indigenous community members' views on the cultural appropriateness of the primary health care (PHC) service in a remote town.	There was considerable gap between the perceptions of PHC providers and Indigenous community members on the provision of culturally appropriate PHC services, with many Indigenous community members not perceiving the service as culturally appropriate.	Cultural awareness training should be developed with local Indigenous community leaders and run throughout the year. Training on culturally appropriate communication with patients should be mandatory for front desk staff and IHWs.<br>Improve the physical space of PHC clinics by displaying local Indigenous artwork.<br>Value AHWs as cultural brokers and actively involve them in healthcare team.
Taylor EV, et al (2020)<br>Multiple states	Indigenous health workforce	Qualitative<br>Case study	32 participants - 24 Indigenous and non-Indigenous hospital staff, 5 Indigenous people with cancer and 3 family members	Analyses Indigenous workforce policies and strategies from two health services, which were recognised in a national study as providing innovative services for Indigenous cancer patients and families.	Eight themes were identified from the way the two services supported their Indigenous workforce: strong leadership, proactive employment strategies, an Indigenous Health Unit, an Indigenous Liaison Officer (ILO), integration of the ILO within teams, professional development opportunities, supportive work environments, and a culture of respect. These themes were present in both	Embed a commitment to improving Indigenous health outcomes within policies and processes, including a Reconciliation Action Plan and an Indigenous Employment Strategy.<br>Develop understanding of the current Indigenous workforce to inform future employment and professional development opportunities.<br>Increase engagement with Indigenous staff, patients and communities and seek their input.<br>Assess recruitment processes and

Author (Year) Location	Category	Method and Study design	Study population	Study aim	Key findings	Recommendations
					services, leading to positive outcomes for both Indigenous patients and staff.	remove barriers for Indigenous jobseekers.
Taylor, E. V., et al. (2019)<br>Not applicable	Student workforce	Systematic review	Systematic review of 26 articles	This review identified<br>i) the factors affecting the retention of Indigenous students across all tertiary health disciplines, and<br>ii) strategies that support Indigenous students to continue and successfully complete their studies	Key factors students reported as affecting retention were family and peer support; competing obligations; academic preparation and prior educational experiences; access to the Indigenous Student Support Centre; financial hardship; and racism and discrimination.<br>The most successful retention strategies implemented by health faculties to improve retention were multi-layered and included: culturally appropriate recruitment and selection processes; comprehensive orientation and pre-entry programs; building a supportive and enabling school culture; appointing Indigenous academics; embedding Indigenous content throughout the curriculum; developing mentoring and tutoring programs.	To better support Indigenous students, universities need to develop programs that incorporate the whole of student life; starting with recruitment and selection, continuing through pre-entry preparation and orientation, and including cultural, academic, social and economic support while at university, as well as considering point of departure.<br>Universities need to facilitate opportunities for Indigenous students to meet and connect through mentoring programs, student networks and gatherings.
Taylor, S., et al. (2018)<br>QLD	Student workforce	Qualitative<br>Focus groups	Fifteen undergraduate health students (second year+) on rural clinical placements	Explores health care students' understanding of the role of the pharmacist in compounding medications to improve health outcomes for patients in rural and remote health care services.	Students from multiple health disciplines valued the learning opportunity and the hands-on aspect of the compounding workshop. They reported improved knowledge and understanding of this specialized activity and recognized the application to practice, particularly in a rural,	These compounding workshops are an effective method for facilitating interprofessional collaborative practice. Future research should explore further the benefits of engaging students in specialty practices in both rural and urban settings to determine whether the experience, knowledge, and understanding gained will impact on future practice.

Author (Year) Location	Category	Method and Study design	Study population	Study aim	Key findings	Recommendations
					remote, and Indigenous context.	
Thackrah, R. D., et al. (2021) WA	Health workforce	Qualitative Interviews	16 participants - 14 non-Indigenous midwives, 2 Indigenous midwives	Reports on early career midwives' observations, experiences and responses to racism in maternity settings. Midwives had previously completed university training aimed at increasing their cultural capabilities.	Positive impact of well-designed Indigenous content in training programs and placements, learnings applied in workplace years later. Non-Indigenous participants aware and responsive to casual racism but not recognising institutional racism. Indigenous midwives experienced and were attuned to racism.	Raise awareness of institutional racism in healthcare training to increase recognition and appropriate responses. Importance of Indigenous health professionals and mentors in providing training and professional development.
Thackrah, R. D., et al. (2013) WA	Student workforce	Mixed methods Questionnaire and interviews	15 midwifery students	Explores midwifery students' responses to Indigenous content in their program.	While most midwifery students were receptive to Indigenous content in their program and acknowledged its importance to clinical practice, diversity of attitudes and experiences sometimes created tensions in the classroom. Unresolved issues around race and racism were observed and were rarely challenged due to classroom dynamics.	Further research is required to establish whether Indigenous content covered in the course is retained and applied in clinical settings. Integration of content throughout the program and enhanced opportunities for clinical practice with Indigenous women may assist this process.
Thackrah, R. D., et al. (2015) WA	Student workforce	Quantitative Survey	44 students in a direct entry, undergraduate midwifery program	Explores undergraduate midwifery students' knowledge and attitudes towards Indigenous people, and the impact of Indigenous content in their program.	A positive shift in first year students' knowledge and attitudes towards Indigenous people was observed, with evidence that teaching in the unit was largely responsible for this shift. However, the gains were not sustained, with a significant decline in knowledge, attitude and impact of the content observed in subsequent years. All students indicated a strong	Additional midwifery-specific Indigenous content related to pregnancy and birthing, and recognition of strong student interest in clinical placements in Indigenous settings provide opportunities for future curriculum development.

Author (Year) Location	Category	Method and Study design	Study population	Study aim	Key findings	Recommendations
					interest in more clinical exposure to Indigenous settings.	
Thackrah, R. D., et al. (2017) WA	Student workforce	Qualitative Interviews	12 health science students and recent graduates	Explores the experiences of health science students and recent graduates who spent up to 4 weeks on placement in a remote community.	Factors which contributed to positive learning experiences included pre-placement cultural training to build understanding of the local Indigenous community, peer support, community engagement, cultural exchanges and interprofessional collaboration. An Indigenous mentor was vital to students' understanding of the social and cultural dynamics in the practice setting. Challenges included the logistics of supervision in remote locations and workloads.	Longitudinal studies are required to build an evidence base in this area. Further research into the impact of service learning on remote communities is needed to ensure that mutual benefits are derived from placements and community needs prioritised.
Thackrah, R. D., et al. (2019) WA	Student workforce	Quantitative Interviews	7 health science graduates who had previously completed a remote placement in 2013/4 (Stage One)	This study reports on the second stage of an investigation into the longer-term impact of remote placements. This follow-up study explores the impact of a remote placement on professional practice and employment decision-making amongst a subset of the original cohort.	The remote placement had an enduring effect on participants' personal and professional growth. The majority were employed in rural settings. All participants actively applied clinical and cultural learnings acquired on placement to their professional practice. Rural job security, professional support and opportunities for professional development were all influences on continuing rural practice.	Qualitative, longitudinal studies of health professionals are required to explore employment decision-making and the contribution of rural placements to a more stable and culturally respectful rural workforce.
Thackrah, R. D., et al. (2013) WA	Student workforce	Mixed methods Questionnaires and classroom observations	12 non-Indigenous midwifery students	The emotional responses of students undertaking a new, compulsory unit on Indigenous cultures and	A spectrum of emotional responses was identified. While stereotypes were challenged and perceptions altered	Content on Indigenous health and cultures needs to extend beyond one unit in a course. Learning and knowledge must be carefully

Author (Year) Location	Category	Method and Study design	Study population	Study aim	Key findings	Recommendations
				health were investigated as part of a broader study looking at culturally secure practice in midwifery education and service provision for Indigenous women.	because of the content, issues surrounding racism remained unresolved, with some students expressing dismay at the attitudes of their peers.	integrated across training and developed to maximise understanding and ensure that unresolved issues are addressed.
Thackrah, R. D., et al. (2014)<br>WA	Student workforce	Qualitative Interviews	7 midwifery students	Describes midwifery students' experiences and reflections on a short remote Indigenous clinical placement.	The remote clinical placement was highly valued by all participants and recommended to others.<br>Highlights included connections made with community members and cultural knowledge learned experientially.<br>Challenges included geographic and professional isolation and the complexities of health care delivery in remote settings.<br>All students recognised the transferability of the knowledge and skills acquired to urban settings; some had incorporated these learnings into clinical practice.	Cultural immersion programs which provide opportunities for students to learn from and interact with community members in supervised practice settings can deliver rich learning experiences that are not acquired in classroom settings and can have real and immediate impact on clinical practice.
Thackrah, R. D., et al. (2015)<br>WA	Student workforce	Qualitative Interviews	7 midwifery students	Describes midwifery students' insights on promoting health to Indigenous women living remotely following a supervised clinical placement.	Students observed that high rates of participation in a breast screening program were achieved due to the informal provision of culturally relevant information and support.<br>Opportunistic encounters in communities enabled sexual health messages to be delivered in less formal settings.<br>The importance of Indigenous Health Workers, culturally respectful approaches to sensitive women's business,	A short duration remote placement enabled students to understand local contexts, cultural protocols, community needs and barriers to care which had not been gained in prior placements.

Author (Year) Location	Category	Method and Study design	Study population	Study aim	Key findings	Recommendations
					the use of local language and pictorial representations of information, was recognised.	
Thackrah, R. D., et al. (2018) WA	Student workforce	Mixed methods Questionnaires, classroom observations and interviews	44 undergraduate midwifery students	Explores the impacts of an Indigenous health unit and remote clinical placements on knowledge acquisition and attitude change among midwifery students.	A unit with substantial Indigenous input and which privileged these voices enhanced knowledge and shifted attitudes in a positive direction; however, immediate gains diminished over time. Remote placements had a profound effect on student learning. Exposure to Indigenous Australians in classrooms and communities helped dispel stereotypes and challenge assumptions.	Vertical integration of Indigenous content in university courses and enhanced opportunities to apply learnings in community settings are essential for gains to be maximized.
Tsey, K., et al. (2014) Multiple states	Indigenous health workforce	Mixed methods Interviews, workshops, questionnaires, focus groups and participant observations	25 participants - 9 Male Health Module trainers, 16 health workers who attended the Male Health Module workshop	Evaluates the pilot phase of an Indigenous Male Health Module, an educational program to support Indigenous health workers utilising a gender-based approach to increase participation of Indigenous males in health care.	There was overall enthusiasm for the Module. As well as potential to enhance access to health services for men, the Module promotes health worker empowerment and wellbeing Male and female health workers working in partnership was considered necessary.	Partnerships between curriculum developers, training providers and relevant Indigenous male support networks are needed to make the course accessible to men. Male-friendly health programs can improve men's health-seeking behaviours.
Tyrrell, M. S., et al. (2020) National	Health workforce	Quantitative Survey	547 health professionals (allied health, medical, nursing) across all levels of remoteness	Identifies what motivates health professionals to work in a very remote Indigenous community for more than 3 years.	Developed 14 health practitioner work motivation scales including 8 sensitive to very remote Indigenous community work choice. Developed likelihood estimator for health professionals continuing to work in remote Indigenous communities for longer than 3 years.	Four motivation subscale scores together provided a significant estimate of likelihood of a practitioner having more than 3 years' work experience in a very remote Indigenous community, compared with no such experience (retention predictive model). Further research is required to confirm, further validate and refine the scales.

Author (Year) Location	Category	Method and Study design	Study population	Study aim	Key findings	Recommendations
Webster, S., et al. (2010) NSW	Student workforce	Mixed methods Questionnaire (closed-ended and open-ended questions)	8 second-year nursing students	Explores how a primary health care clinical placement in Indigenous communities can provide nursing students with a rich and varied learning experience and insights into complex aspects of rural life including Indigenous health.	Clinical experience in rural areas can positively influence attitudes, preparedness for practice and engage students on many levels, deepening their understanding of rural communities and related rural health issues.	The value of rural placements as a method for increasing nursing student's practical experience should be promoted.<br>There is room for improvement in effective preparation and support of students in their placement.<br>Opportunities to learn about culturally appropriate health care in Indigenous settings need to be further developed to engage students in the learning process
Wright, J. R., et al. (2014) Vic	Student workforce	Mixed methods Survey and focus groups	69 medical students who took part in a 3-week Rural Health Module	To determine whether a short-term placement of metropolitan medical students in a rural environment can improve their knowledge of, and change their attitudes to, rural health issues.	The focus groups identified five main themes in rural health care: access; teamwork, models of care and generalist practice; overlapping relationships; indigenous health; and working in a rural career. In all five areas, a change was seen in the depth of knowledge students had about these issues and in students' attitudes towards rural health care. The questionnaires also showed a significant shift in the students' appreciation of, and positivity towards, rural health issues.	None.
Zhao, Y., et al. (2017) NT	Health workforce	Quantitative NT Department of Health payroll and financial dataset analysis	Staff employed in 54 remote clinics, 2004–2015 (including nurses, Aboriginal Health Practitioners (AHPs) and staff in administrative and logistic roles)	Describes temporal changes in workforce supply in government-operated clinics occurred in remote NT communities over a period where there was a substantial increase in health funding. Overall increases in workforce supply were affected by	Overall increases in workforce supply occurred between 2004 and 2015, especially for administrative and logistic positions. Supply of nurses and AHPs increased from an average 2.6 to 3.2 FTE per clinic, although supply of AHPs has declined. Only a modest increase in nurse and AHP FTE supply occurred in remote	More robust health service models are vital to better support the supply and retention of resident health staff.<br>It is critical to invest in developing stronger career pathways for AHPs and nurses and to implement more robust health service models

Author (Year) Location	Category	Method and Study design	Study population	Study aim	Key findings	Recommendations
				continuing very high turnover of nurses and AHPs, compounded by declines in AHP supply.	communities, with most remote health clinics unable to sustain initial increases in NT government employed nurses and AHPs.	

Abbreviations: AHP = Aboriginal Health Practitioner; AHW = Aboriginal Health Worker; AMHW = Aboriginal mental health worker; CBT = Cognitive Behavioural Therapy; CMH = community mental health; d-SEWB = digital social and emotional wellbeing; eMH = e-Mental Health; FTE = full-time equivalent; IHW = Indigenous Health Worker; ILO = Indigenous Liaison Officer; NSW = New South Wales; NT = Northern Territory; PEPA = Program of Experience in the Palliative Approach; PHC = Primary health care; QLD = Queensland; RAN = Remote area nurse; SW = Social Worker; Vic = Victoria; WA = Western Australia
